# Redox-Driven Precision Medicine for Life-Course Prevention of Cardiovascular–Kidney–Metabolic Syndrome

**DOI:** 10.3390/antiox15020221

**Published:** 2026-02-08

**Authors:** Chien-Ning Hsu, You-Lin Tain

**Affiliations:** 1 Department of Pharmacy, Kaohsiung Municipal Ta-Tung Hospital, Kaohsiung 801, Taiwan; cnhsu@cgmh.org.tw; 2 Department of Pharmacy, Kaohsiung Chang Gung Memorial Hospital, Kaohsiung 833, Taiwan; 3 School of Pharmacy, Kaohsiung Medical University, Kaohsiung 807, Taiwan; 4 Department of Pediatrics, Kaohsiung Chang Gung Memorial Hospital, Kaohsiung 833, Taiwan; 5 College of Medicine, Chang Gung University, Taoyuan 333, Taiwan; 6 Doctoral Program of Clinical and Experimental Medicine, National Sun Yat-sen University, Kaohsiung 804, Taiwan

**Keywords:** oxidative stress, cardiovascular-kidney-metabolic syndrome, developmental origins of health and disease (DOHaD), precision medicine, antioxidants, nitric oxide

## Abstract

Accumulating evidence recognizes cardiovascular–kidney–metabolic syndrome (CKMS) as a life-course disorder arising from dynamic and maladaptive interactions among the heart, vasculature, kidneys, liver, and pancreas. Beyond a late-onset clinical entity, CKMS susceptibility is increasingly understood to be programmed during critical developmental periods. Redox imbalance has emerged as a central integrative mechanism in this process, functioning as a mechanistic interface through which adverse early-life environments translate into persistent multi-organ vulnerability. Perturbation of the reactive oxygen species–nitric oxide axis during development disrupts organogenesis, vascular maturation, and metabolic regulation, resulting in enduring structural and functional alterations that predispose individuals to hypertension, metabolic dysfunction, and chronic kidney disease. These insights position redox biology not merely as a pathogenic mechanism but as a strategic entry point for precision intervention. Addressing the escalating global burden of CKMS requires a paradigm shift toward redox-driven precision medicine. This framework integrates biologically informed phenotyping, life-course–based risk stratification, early precision prevention through developmental reprogramming, and phenotype-guided therapeutics to stabilize interconnected organ networks. Transitioning from reactive, fragmented care to a proactive, systems-oriented approach offers a transformative opportunity to interrupt intergenerational risk transmission and achieve durable improvements in cardiovascular–kidney–metabolic health across the lifespan.

## 1. Introduction

Cardiovascular disease (CVD) remains the leading cause of death worldwide, accounting for more than 19 million deaths annually [1]. A growing body of evidence indicates that major metabolic disorders—including obesity, type 2 diabetes, and chronic kidney disease (CKD)—do not merely coexist as independent cardiovascular risk factors but are biologically interconnected through bidirectional and self-reinforcing pathways. These interactions form the basis of the cardiovascular–kidney–metabolic syndrome (CKMS), a systemic disorder characterized by coordinated dysfunction across the heart, vasculature, kidney, liver, and pancreas [2]. Within this networked disease state, pathological processes propagate across organs, accelerating tissue injury and generating a level of cardiovascular risk that exceeds the additive effects of individual conditions. Epidemiological estimates suggesting that nearly 90% of adults in the United States exhibit features of CKMS underscore the urgent need for strategies that enable early detection, prevention, and mechanistic modeling of inter-organ crosstalk to mitigate the global burden of CVD [3].

The integrated and heterogeneous nature of CKMS poses substantial challenges to conventional disease management frameworks. Its broad phenotypic spectrum and extensive organ-to-organ connectivity limit the effectiveness of uniform therapeutic approaches, highlighting the necessity for precision medicine strategies that account for individual disease trajectories. Importantly, susceptibility to CKMS does not arise solely from adult lifestyle or genetic predisposition. Accumulating experimental and clinical evidence demonstrates that adverse environmental exposures during gestation and early postnatal life program long-term vulnerability to metabolic, renal, and cardiovascular disorders [4,5,6,7]. Within the developmental origins of health and disease (DOHaD) paradigm, fetal adaptations to early-life cues may confer survival advantages in the short term while establishing latent risks for chronic disease later in life [8]. Importantly, such developmental programming is not irreversible. Interventions applied during sensitive developmental windows—prior to the manifestation of overt disease—may modify disease susceptibility, a concept referred to as reprogramming [9]. Viewing CKMS as a life-course disorder with origins before birth reframes prevention, rather than treatment, as a central strategy for reducing its long-term impact.

Oxidative stress has emerged as a pivotal mechanistic driver within the DOHaD framework, acting as a redox-sensitive nexus that links adverse early-life environments to the later development of CKMS [10,11,12]. Under physiological conditions, precisely regulated reactive oxygen species (ROS) and nitric oxide (NO) serve essential signaling roles in placental vascularization, organogenesis, and epigenetic regulation [13]. Maternal insults—such as hypoxia, nutritional imbalance, obesity, and exposure to environmental toxicants—disrupt this balance, shifting redox homeostasis toward ROS/NO disequilibrium. Excess superoxide rapidly quenches NO to form peroxynitrite, initiating endothelial nitric oxide synthase uncoupling, mitochondrial dysfunction, and impaired bioenergetics during critical periods of development. These redox-mediated disturbances compromise nephron endowment, vascular responsiveness, and metabolic set-points, thereby predisposing offspring to hypertension, CKD, insulin resistance, and CVD in later life [14,15,16,17]. The Janus-faced biology of ROS and NO—indispensable for physiological signaling at basal levels yet pathogenic when diverted toward peroxynitrite-driven injury—underscores that oxidative stress is not merely a marker of damage but a causal mechanism of developmental programming [18,19,20].

Accordingly, effective prevention of CKMS requires a precision medicine paradigm that integrates multi-omics, systems biology, and network-based approaches to selectively interrupt pathogenic redox circuits while preserving homeostatic signaling across the CKMS continuum [21,22]. Conceptually, this approach resembles the maintenance of an interconnected power grid: targeted, timely correction of a single redox “fault” during early development may avert cascading system-wide failure culminating in irreversible multiorgan disease. This review synthesizes emerging clinical and experimental evidence defining the role of oxidative stress in CKMS, with particular emphasis on precision prevention. Insights from animal models have been instrumental in elucidating core developmental mechanisms and identifying redox-sensitive targets amenable to early intervention [12,23,24]. Integrating these mechanistic insights with human data is essential not only for refining oxidative stress-related biomarkers but also for informing innovative reprogramming strategies aimed at mitigating or reversing adverse developmental trajectories, thereby addressing the escalating global burden of CVD and related metabolic–kidney disorders.

## 2. Materials and Methods

Given the conceptual breadth and methodological heterogeneity of the available evidence, we adopted a narrative review approach rather than a systematic or scoping review to enable an integrative synthesis of emerging concepts across clinical medicine, developmental biology, redox signaling, and environmental health. A comprehensive literature search was conducted to identify relevant English-language studies published between January 2000 and December 2025. Both experimental and clinical investigations were retrieved from major scientific databases, including MEDLINE, Embase, and the Cochrane Library, with emphasis placed on full-text original research and high-quality reviews. The search strategy incorporated keywords related to oxidative stress and redox biology (“oxidative stress,” “reactive oxygen species,” “reactive nitrogen species,” “nitric oxide,” “antioxidants”), precision medicine (“omics,” “biomarker,” “targeted therapy”), and CKMS-related phenotypes (“metabolic syndrome,” “obesity,” “chronic kidney disease,” “cardiovascular disease,” “hypertension,” “dyslipidemia,” “insulin resistance,” “diabetes,” “atherosclerosis,” “heart failure,” and “liver steatosis”), as well as developmental programming within the DOHaD framework (“developmental programming,” “DOHaD,” “life course theory,” “offspring,” “maternal,” “pregnancy,” “lactation,” and “reprogramming”). Reference lists of retrieved articles were manually screened to identify additional pertinent studies. This approach enabled a cohesive appraisal of mechanistic insights and translational evidence relevant to precision prevention of CKMS.

## 3. CKMS Across the Life Course

The conceptualization of the CKMS marks a paradigm shift in chronic disease research, moving beyond isolated organ-based pathologies toward a systemic, multidimensional framework defined by dynamic pathophysiological interdependence [25]. As a network-driven disorder, CKMS is characterized by reciprocal risk amplification, whereby interactions among metabolic, renal, and cardiovascular abnormalities generate outcomes that far exceed the impact of any single condition [26]. For example, while diabetes and CKD independently confer substantial mortality risk, their coexistence precipitates a synergistic deterioration, with markedly elevated long-term mortality rates [27]. Importantly, CKMS is not confined to the cardiovascular–kidney–metabolic axis but extends to other organ systems, including the brain. Systemic low-grade inflammation, endothelial dysfunction, insulin resistance, and renin–angiotensin system dysregulation collectively impair cerebrovascular function and neural homeostasis, increasing susceptibility to cognitive impairment, stroke, and neurodegenerative disorders [28]. These observations underscore CKMS as a multisystem disease with broad organ involvement rather than a condition limited to traditional CKM endpoints. This complexity challenges clinician-scientists to interrogate disease mechanisms across the life course, from early epigenetic influences to the eventual convergence of multi-organ dysfunction in adulthood [29].

To operationalize intervention across this continuum, the CKMS staging framework (Stages 0–4) provides a structured approach that emphasizes prevention at the earliest possible stages [2]. Stage 0 denotes the absence of CKMS risk factors, prioritizing primordial prevention and the preservation of ideal cardiovascular health. Progression to Stage 1 reflects excess or dysfunctional adiposity, often preceding overt metabolic disease. Stage 2 is defined by the emergence of established metabolic and renal risk factors, including hypertension, dyslipidemia, type 2 diabetes, or moderate-to-high-risk CKD. Stage 3 captures subclinical cardiovascular injury, identified through imaging or biomarker evidence, as well as high-risk equivalents such as advanced CKD. Stage 4 represents overt clinical CVD and is further stratified by the presence or absence of kidney failure to accommodate distinct management challenges. Importantly, this staging construct reframes CKMS not as an inevitable late-stage diagnosis but as a progressive disorder amenable to early-life and preclinical intervention.

Despite its clinical utility, CKMS staging alone is insufficient to capture the profound heterogeneity that defines disease trajectories across the life course [27]. Individuals with comparable anthropometric profiles frequently diverge in metabolic, renal, and cardiovascular outcomes, reflecting nonlinear, bidirectional pathways and variable rates of progression that are often established long before overt risk factors become clinically apparent. This variability is particularly evident across populations; for instance, individuals of Asian ancestry develop cardiometabolic complications at lower levels of adiposity [30], a pattern increasingly attributed to early-life determinants of ectopic fat distribution, metabolic programming, and cumulative environmental exposures rather than adult body composition alone [31,32]. Such observations underscore that CKMS heterogeneity is not merely a contemporaneous clinical phenomenon but the downstream expression of distinct biological trajectories shaped by genetic, developmental, behavioral, and social influences over time.

Addressing this complexity requires a decisive shift from late-stage risk stratification toward a life-course–oriented precision prevention paradigm [33]. Rather than relying solely on adult phenotypes or coarse staging metrics that incompletely reflect underlying pathophysiology, effective CKMS prevention must prioritize the identification and modification of susceptibility during critical windows of development, when physiological systems remain plastic and amenable to intervention. Achieving this goal necessitates moving beyond traditional specialty-based silos and adopting a network medicine framework that integrates molecular, metabolic, and developmental processes into individualized risk trajectories. Within this context, early-life prevention becomes foundational rather than ancillary, positioning precision medicine as a proactive strategy to redirect disease pathways before irreversible CKMS is established [34,35,36].

## 4. The Developmental Blueprint: The “Redox Switch” in Early Life

**Figure 1:**
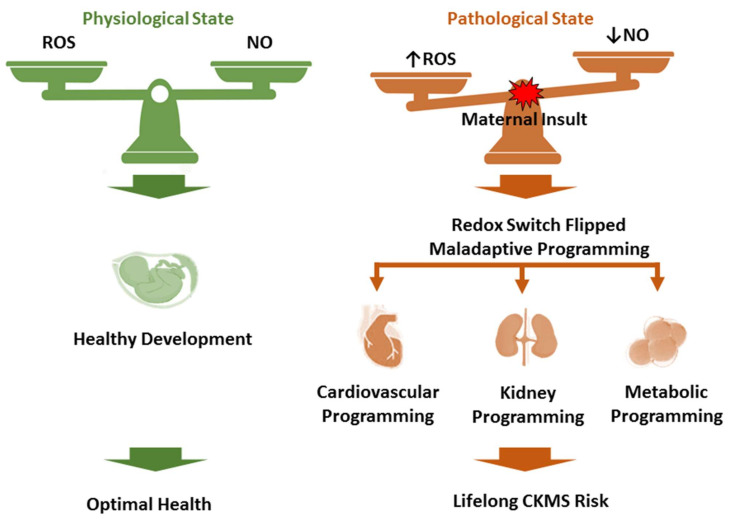
A redox-regulated developmental network connecting early-life exposures to multi-organ dysfunction in cardiovascular–kidney–metabolic syndrome (CKMS).

Conceptualizing CKMS as a life-course disorder highlights that its pathological trajectory is frequently established during critical, stage-specific windows of fetal and early postnatal development, during which cardiometabolic and renal systems exhibit heightened developmental plasticity. Within the DOHaD framework, the maternal–fetal interface operates as a redox-sensitive and context-dependent “switch,” rather than a binary on/off mechanism, in which the tightly regulated balance between ROS and NO governs organogenesis, vascular maturation, and long-term physiological set-points [37,38]. Under normal conditions, controlled ROS signaling is indispensable for cellular differentiation and tissue development [39,40]. In contrast, excessive oxidative stress—defined by ROS production exceeding antioxidant capacity—disrupts this developmental blueprint and drives maladaptive, tissue-specific programming, with particular vulnerability in the renal vasculature, cardiomyocytes, and central neuroendocrine circuits across renal, metabolic, and cardiovascular systems [12,13,14,15] (Figure 1).

### 4.1. Early-Life Insults and Redox Imbalance

This redox switch is commonly shifted toward a pro-oxidant state by adverse early-life exposures, including maternal nutritional imbalance (e.g., high-fat or high-fructose diets) [41,42], maternal illness (e.g., diabetes or CKD) [43,44], placental insufficiency or hypoxia [45,46], pharmacological exposures [47], and environmental toxicants such as dioxins or phthalates [48,49], as demonstrated in rodent models. A unifying mechanism underlying these insults is impairment of the ROS/NO pathway [16,50]. Elevated asymmetric dimethylarginine (ADMA), an endogenous inhibitor of nitric oxide synthase (NOS), reduces NO bioavailability and promotes NOS uncoupling, leading to excess superoxide and peroxynitrite formation [51,52]. Emerging evidence highlights additional molecular pathways underlying these redox disturbances [53], including Nrf2/Keap1 signaling [54], which regulates antioxidant gene expression; NOX isoforms [55], which contribute to controlled ROS generation; and mitochondrial quality control mechanisms [56], which maintain cellular bioenergetics and prevent oxidative injury. This redox disequilibrium induces oxidative damage to DNA, proteins, and lipids, thereby distorting developmental signaling pathways and permanently altering organ structure and function [57].

### 4.2. Kidney Programming

The developing kidney is particularly vulnerable to oxidative stress during the peak period of nephrogenesis [58]. Maternal caloric restriction [59], protein deficiency [60], medication use [61], maternal smoking [62], or exposure to environmental toxicants [48] markedly increases ROS production, impairing ureteric bud branching and reducing nephron endowment. Disruption of the ADMA–NO axis plays a central role, as elevated ADMA suppresses NO synthesis and exacerbates NOS uncoupling, amplifying oxidative injury leading to kidney programming [50]. These redox-driven perturbations result in irreversible structural alterations, including reduced nephron number, glomerular hypertrophy, and tubulointerstitial damage. Functionally, these changes promote glomerular hyperfiltration and compensatory hypertension, establishing a lifelong susceptibility to CKD [63,64].

### 4.3. Metabolic Programming

CKMS frequently originates from excess or dysfunctional adiposity, a process profoundly shaped by early-life redox imbalance [2]. Excessive ROS exposure during prenatal or neonatal periods disrupts adipocyte maturation and induces low-grade inflammation within adipose tissue, favoring visceral and ectopic fat deposition in organs such as the liver and heart [65,66]. This maladaptive metabolic programming impairs insulin receptor signaling, activates pro-inflammatory pathways (e.g., TNF-α, IL-6), and promotes mitochondrial dysfunction, creating a self-reinforcing cycle of oxidative stress and insulin resistance [67]. Over time, these processes accelerate the transition from impaired glucose tolerance to overt type 2 diabetes mellitus. As an active endocrine organ, dysfunctional adipose tissue further propagates systemic inflammation and oxidative stress, contributing to endothelial dysfunction and reinforcing cardiorenal interactions [2].

### 4.4. Cardiovascular Programming

Early-life redox imbalance also exerts lasting effects on cardiovascular development [68]. Excess ROS during cardiac and vascular maturation reduces NO bioavailability, increases arterial stiffness, and impairs endothelial function [14]. Experimental models demonstrate that maternal hypoxia [69] or inflammation [70] induces cardiomyocyte loss, maladaptive cardiac remodeling, and activation of profibrotic pathways such as TGF-β signaling. These structural and molecular alterations establish a pro-atherogenic and pro-fibrotic cardiovascular phenotype, substantially increasing lifetime risk for atherosclerotic CVD and heart failure [71]. Importantly, programmed cardiac vulnerability interacts bidirectionally with kidney dysfunction, amplifying CKMS progression through maladaptive cardiorenal crosstalk [72].

Collectively, these observations underscore oxidative stress and specific molecular redox pathways as integrative, rather than singular, mechanistic drivers of developmental programming in CKMS. Addressing this complexity necessitates a shift toward precision, redox-based prevention strategies. Targeted antioxidant or NO-restoring interventions applied during defined developmental windows of retained redox plasticity have shown promise in experimental models by restoring redox homeostasis and “reprogramming” adverse trajectories [73,74]. By intervening early, it may be possible to interrupt the intergenerational transmission of cardiovascular, renal, and metabolic risk, redefining prevention as a foundational component of CKMS management across the life course.

## 5. Biomarkers for CKMS Across the Lifespan

CKMS is a complex systemic disorder driven by dynamic interactions among metabolic dysregulation, CKD, and cardiovascular dysfunction. Its pronounced heterogeneity across the life course necessitates a shift toward precision prevention supported by stage-specific, mechanism-based biomarkers. To enhance translational relevance, CKMS biomarkers can be pragmatically prioritized according to their current clinical readiness, ranging from clinically implemented markers to research-grade and exploratory multi-omics signatures. Across developmental stages, these biomarkers delineate disease evolution from early-life redox imbalance to progressive cardiometabolic and renal complications in adulthood.

### 5.1. Early-Life and Prenatal Biomarkers

Within the DOHaD framework, the prenatal period represents a critical window during which environmental exposures can durably shape cardiovascular, renal, and metabolic trajectories. Biomarkers of prenatal oxidative stress are central mediators of this developmental programming relevant to CKMS.

NO is essential for maternal–fetal vascular homeostasis and placental perfusion [75]. Under adverse intrauterine conditions, elevated levels of ADMA impair NO bioavailability and promote NOS uncoupling, shifting redox balance toward superoxide generation [76]. Experimental evidence demonstrates that prenatal excess of ADMA induces dose-dependent reductions in nephron endowment [77], thereby predisposing offspring to hypertension and CKD later in life. Clinically, disruption of the ADMA–NO axis is closely associated with pregnancy complications such as preeclampsia [78] and gestational diabetes [79], positioning ADMA as a clinically available but underutilized biomarker with strong mechanistic and translational relevance to early CKMS programming.

Because ROS are inherently transient, stable markers of oxidative damage provide more reliable indicators of prenatal redox stress. F_2_-isoprostanes, reflecting lipid peroxidation [80,81], and 8-hydroxy-2′-deoxyguanosine, capturing oxidative DNA damage [82], have both been linked to experimental and clinical models of CKMS programming [41,83,84]. These biomarkers are best classified as research-grade, as their broader clinical adoption remains limited by pre-analytical stability, assay standardization, and cost considerations. These markers are frequently accompanied by epigenetic alterations that increase long-term susceptibility to metabolic dysfunction and CKD. Circulating microRNAs [85], particularly hypoxia-responsive miR-210 [86], represent emerging research-grade biomarkers that capture redox-sensitive transcriptional and mitochondrial adaptations but currently lack harmonized analytical platforms and reference ranges. Together, these prenatal biomarkers enable early identification of maladaptive trajectories long before clinical manifestations of CKMS emerge.

### 5.2. Pediatric Biomarkers: Lessons from Children with CKD

In pediatric CKD, oxidative stress emerges early and serves as a unifying mechanism linking kidney injury to cardiovascular and metabolic dysfunction [33,87]. Excessive ROS production, coupled with impaired antioxidant defenses, contributes to subclinical vascular and cardiac damage during childhood.

Oxidative modification of lipoproteins, particularly oxidized low-density lipoprotein, has been associated with hypertension and left ventricular hypertrophy in children with CKD [88], indicating early cardiac remodeling prior to overt CVD. Beyond lipid oxidation, chronic oxidative burden interacts with inflammatory and metabolic pathways, promoting endothelial activation, arterial stiffness, renal fibrosis, and metabolic dysregulation [89,90]. This interconnected pathology exemplifies the multisystem nature of pediatric CKMS.

Gut microbiota–derived uremic toxins further amplify oxidative stress–mediated injury. Elevated levels of TMAO and tryptophan-derived metabolites, including indoxyl sulfate and p-cresyl sulfate, have been linked to impaired kidney function and early vascular remodeling in pediatric CKD [91,92]. While analytically measurable, these metabolites currently remain research-grade biomarkers due to inter-individual variability, dietary confounding, and limited standardization across platforms. Despite increasing recognition of these pathways, no single oxidative stress marker sufficiently captures disease complexity [93]. Accordingly, integrated biomarker panels that combine clinically available renal markers with research-grade oxidative and metabolic indicators offer the most realistic strategy for early pediatric risk stratification.

### 5.3. Adult Biomarkers: Staging and Precision Management

In adults, biomarkers constitute the biological foundation of CKMS staging by reflecting cumulative metabolic burden and multisystem injury [2]. Early stages are characterized by markers of metabolic stress and insulin resistance, including glycemic indices and composite lipid–glucose metrics that more accurately capture cardiometabolic risk than isolated parameters [94,95].

With disease progression, biomarkers increasingly signify subclinical cardiovascular injury and maladaptive cardiac remodeling. Coronary artery calcium scoring, high-sensitivity cardiac troponin, and natriuretic peptides identify high-risk phenotypes prone to heart failure [96,97]. Among these, high-sensitivity cardiac troponin represents a clinically ready biomarker that enables detection of silent myocardial injury in CKMS. Concurrently, kidney-related biomarkers remain central to CKMS progression. Estimated glomerular filtration rate (eGFR) and urine albumin-to-creatinine ratio provide foundational assessments of renal health [98], while albuminuria independently predicts cardiovascular events [99]. These measures constitute the core clinically actionable biomarkers for CKMS staging and longitudinal monitoring. Injury and inflammatory markers such as KIM-1, TNFR1, TNFR2, and FGF23 further link renal dysfunction to vascular remodeling and left ventricular hypertrophy [100,101,102,103], but are currently limited to specialized or research settings due to assay availability and cost.

Advances in multi-omics technologies have expanded biomarker discovery toward system-level signatures [104]. Gut-derived metabolites, lipidomic profiles, and advanced glycation end products integrate dietary exposure, metabolic flux, mitochondrial dysfunction, and biological aging, offering mechanistic insight into cardiometabolic vulnerability [105,106]. These multi-omics approaches remain exploratory, facing substantial translational barriers including high cost, batch effects, bioinformatic complexity, and limited external validation. Recent genomic studies reveal that much of the so-called “junk DNA” produces long non-coding RNAs (lncRNAs), which regulate gene expression, modulate microRNA–mRNA interactions, and serve as emerging prognostic biomarkers and therapeutic targets in kidney disease [107]. Collectively, these findings underscore CKMS as a systems-level disorder that cannot be adequately captured by any single biomarker. A tiered, life-course–oriented biomarker strategy therefore provides a rational framework for precision prevention, balancing immediate clinical applicability with future translational innovation.

## 6. Precision Medicine vs. CKMS: The Need for a New Paradigm

**Figure 2:**
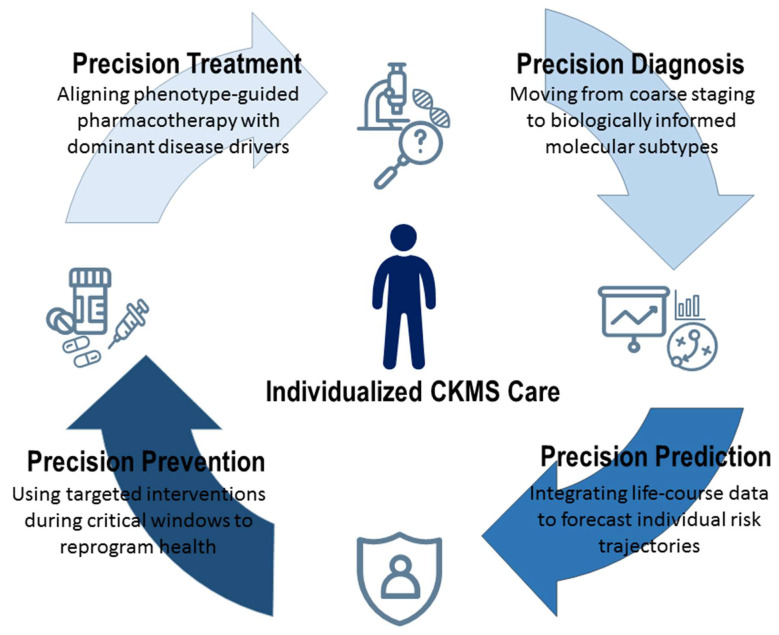
Four core dimensions of precision medicine form an integrated framework for advancing individualized cardiovascular–kidney–metabolic syndrome (CKMS) care.

While conventional staging systems provide a useful clinical framework [2], they do not fully reflect the marked interindividual variability and life-course–dependent trajectories inherent to CKMS. Precision medicine therefore offers a unifying paradigm that enables precision diagnosis through refined phenotyping of multisystem involvement, precision prediction by integrating genetic susceptibility, environmental exposures, and longitudinal risk profiles, precision prevention via early identification of vulnerable individuals during critical developmental windows, and precision treatment through personalized therapeutic strategies tailored to an individual’s biological, environmental, and lifestyle context [34,35,36]. Together, these four dimensions establish a comprehensive foundation for advancing CKMS care beyond uniform management toward truly individualized clinical decision-making (Figure 2).

### 6.1. Precision Diagnosis: Beyond Conventional Staging

Precision diagnosis represents a fundamental shift in the management of CKMS, moving beyond symptom-based categorization toward biologically informed disease characterization through the integration of clinical, molecular, and environmental data. The AHA CKMS staging construct provides an essential diagnostic scaffold [2], with precision becoming particularly salient at Stage 3, where subclinical CVD is detected using advanced imaging and biomarkers, including coronary artery calcium scoring, N-terminal pro-B-type natriuretic peptide (NT-proBNP), and high-sensitivity cardiac troponin (hs-cTn). Identification of these latent phenotypes enables early recognition of individuals at disproportionately high cardiometabolic and renal risk, well before the onset of overt clinical events.

Obesity provides a paradigmatic example of how precision diagnosis reshapes CKMS-related risk stratification. Advances in whole-exome and whole-genome sequencing have enabled earlier identification of syndromic and monogenic forms of childhood obesity, including Prader–Willi syndrome, Bardet–Biedl syndrome, and defects in the leptin–melanocortin pathway [108,109]. Early genetic diagnosis not only facilitates tailored clinical management but also reframes obesity as a biologically driven condition rather than solely a behavioral disorder, thereby reducing stigma and supporting mechanism-based intervention.

A representative illustration of precision diagnosis in CKMS-associated kidney disease is provided by the molecular reclassification of CKD proposed by Reznichenko et al. [110]. This framework transcends conventional GFR-based staging by integrating transcriptomic profiling of kidney biopsies with machine learning–based self-organizing maps, revealing four distinct molecular phenotypes—Gold, Olive, Plum, and Blue—each characterized by divergent pathogenic pathways, including inflammation and fibrosis, mitochondrial dysfunction, and altered protein synthesis. Notably, the CKD-Gold phenotype demonstrated the highest risk of progression to kidney failure, underscoring the prognostic superiority of molecular subtyping over traditional clinical or histopathological classifications. Importantly, concordant molecular signatures were detectable in the urine proteome, highlighting the feasibility of noninvasive, mechanism-based diagnosis and longitudinal monitoring.

Beyond conventional staging, precision diagnosis in CKMS increasingly relies on molecular subtyping enabled by multi-omics technologies. Genomic risk stratification using polygenic risk scores identifies individuals at the extremes of inherited susceptibility, while proteomic and transcriptomic profiling refines kidney and cardiovascular phenotypes associated with differential progression risk. Metabolomic signatures, including gut-derived metabolites such as TMAO, further capture maladaptive cardiometabolic–kidney crosstalk and enhance biological resolution [111].

Digital twin technologies exemplify the next evolution of precision diagnosis in CKMS by generating physiologically faithful virtual representations of individual patients through the integration of multimodal data, including wearable sensors, electronic health records, imaging, and omics [35,112]. In cardiovascular medicine, digital twins enable simulation of interventions such as atrial fibrillation ablation, transcatheter aortic valve replacement, and coronary procedures, thereby optimizing procedural planning and predicting patient-specific outcomes [113]. In nephrology, incorporation of molecular CKD subtypes, including CKD-Gold and CKD-Blue, allows kidney digital twins to model individualized disease trajectories and pharmacologic responses [110,114]. Metabolic management is similarly enhanced through real-time feedback systems and predictive models derived from continuous glucose monitoring and gut microbiome data, supporting personalized nutrition and diabetes reversal strategies [115]. In addition, synthetic data generation and in silico clinical trials reduce patient burden while improving representation of rare or under-studied populations.

Collectively, this integrated diagnostic paradigm reframes CKMS as a dynamic, systemic disorder and establishes the foundation for truly individualized, mechanism-based prevention and treatment pathways.

### 6.2. Precision Prediction: The Life-Course Perspective

Precision prediction in CKMS extends beyond population-averaged risk estimates toward individualized assessments that span the entire life course, from early development to late adulthood. This framework integrates longitudinal risk equations, molecular biomarkers, and advanced computational technologies to capture the dynamic and cumulative nature of cardio-kidney-metabolic risk.

#### 6.2.1. The Life-Course Framework: Origins in Utero and Childhood

CKM health is programmed during critical early-life windows. Prenatal oxidative stress and adverse intrauterine environments can permanently reduce nephron endowment, predisposing individuals to hypertension and CKD later in life. Consequently, pediatric-stage primary prevention emphasizes identification of early insults, including congenital anomalies of the kidney and urinary tract (CAKUT) and complications related to preterm birth [116,117]. Screening during childhood and adolescence is essential, as CKMS risk factors—such as elevated blood pressure and excess adiposity—frequently track into adulthood and are associated with subclinical organ damage by midlife [118]. In several Asian countries, mass urinary screening programs for childhood proteinuria have been widely implemented [119,120,121], although their effectiveness remains debated globally [122]. Precision prediction at this stage relies on pediatric-specific tools, including the full age spectrum eGFR equation for kidney function assessment [123] and ambulatory blood pressure monitoring to detect masked hypertension [124]. Despite the substantial public health benefits of early CKD identification, many health systems still lack robust surveillance infrastructures to support systematic monitoring [125].

#### 6.2.2. The PREVENT Equations: A Milestone in Precision Prediction

The American Heart Association’s Predicting Risk of CVD Events (PREVENT) equations represent a major advance in adult precision prediction for individuals aged 30–79 years [27]. By incorporating a life-course perspective through 30-year risk estimation beginning at age 30, PREVENT mitigates false reassurance among younger adults with low short-term but high lifetime cardiovascular risk. The equations integrate kidney function via eGFR as a core predictor, acknowledging CKD as a potent amplifier of cardiovascular risk, and disaggregate outcomes by estimating both total cardiovascular disease and heart failure risk separately—the latter often representing the earliest manifestation in diabetes or CKD [126,127]. Importantly, PREVENT adopts a race-free methodology, replacing race-based adjustments with race-free eGFR calculations and shifting emphasis toward social determinants of health. Consistent with a life-course approach, the equations optionally incorporate the Social Deprivation Index to contextualize risk and support more equitable, tailored intervention strategies [128].

#### 6.2.3. Enhancing Prediction Through Molecular Biomarkers and Omics

Precision prediction is further refined through integration of traditional risk factors with multi-omics signatures and next-generation biomarkers. Circulating markers of subclinical organ stress, including NT-proBNP and hs-cTn, outperform conventional models in predicting heart failure and mortality. Lipidomic profiles [105,106], particularly plasma ceramides and acylcarnitines, have emerged as robust independent predictors of major adverse cardiovascular events. In nephrology, transcriptomic profiling of kidney tissue has identified molecular clusters, such as CKD-Gold [110], that predict rapid progression to kidney failure more accurately than histopathologic classifications alone.

#### 6.2.4. Technological Frontiers: Digital Twins and AI

The future of precision prediction lies in the convergence of artificial intelligence (AI) and digital twin technologies. Digital twins provide probabilistic forecasting of disease trajectories and therapeutic responses, enabling individualized prediction of treatment effects for agents such as SGLT2 inhibitors and GLP-1 receptor agonists, thereby reducing reliance on trial-and-error clinical decision-making [129]. While AI-driven analysis of high-dimensional data and digital twin frameworks offer promising approaches for integrating multi-omics and redox biomarker information, their application in CKMS and redox-guided therapy remains largely preclinical and exploratory. To date, no fully validated examples exist for the use of redox biomarkers in clinical Digital Twin platforms [130].

**Figure 3:**
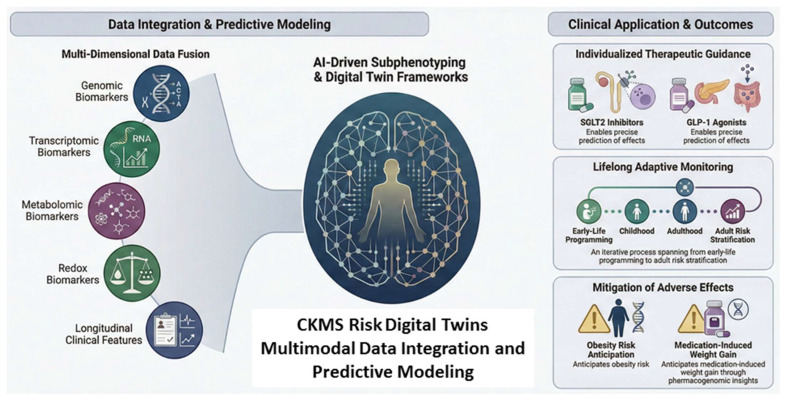
Conceptual framework of digital twin–based cardiovascular–kidney–metabolic syndrome (CKMS) risk stratification through multimodal data integration.

Emerging AI platforms integrate multi-dimensional clinical, biochemical, and molecular data to identify high-risk subphenotypes in CKMS [131,132]. For instance, AI and machine learning tools can combine multi-omics and longitudinal clinical features to enhance prediction of renal and cardiovascular outcomes, supporting precision risk stratification and tailored interventions [132,133]. Conceptually, these systems integrate genomic, transcriptomic, metabolomic, and functional biomarkers—including redox and oxidative stress parameters—allowing probabilistic forecasting of disease trajectories. Figure 3 illustrates this framework, demonstrating how multi-omics and clinical data are combined via AI-driven models to guide individualized monitoring and therapeutic decisions.

Collectively, precision prediction in CKMS is a lifelong, adaptive process starting from early-life programming and extending through adult risk stratification with PREVENT, enriched by molecular subphenotyping, social determinants of health, and AI-enabled monitoring. We explicitly highlight this as a future direction, emphasizing the potential for longitudinal biomarker monitoring, AI-based predictive modeling, and individualized interventions, while acknowledging that translation to routine clinical practice requires further validation. This paradigm also anticipates obesity risk and treatment-related adverse effects [134], as pharmacogenomic studies demonstrate that genetic variants influence susceptibility to medication-induced weight gain [107].

### 6.3. Precision Prevention: Reprogramming the Trajectory

Precision prevention integrates biological and environmental data to optimize early interventions aimed at maintaining health and attenuating disease progression across the CKMS continuum. Accumulating preclinical evidence suggests that adverse cardiometabolic and renal trajectories can be durably “reprogrammed” during sensitive developmental windows through targeted antioxidant strategies, thereby potentially interrupting the intergenerational transmission of CKM risk [13,69].

#### 6.3.1. Antioxidants as Reprogramming Interventions

Central to developmental reprogramming is restoration of the NO pathway, a key regulator of vascular function, nephrogenesis, and redox homeostasis [135]. This conceptually distinct reprogramming strategy contrasts with the largely unsuccessful paradigm of late-life, non-specific antioxidant supplementation in adults (e.g., vitamins E and C), which has shown limited efficacy in cardiovascular and renal outcome trials. Experimental studies demonstrate that diverse antioxidant interventions converge on this shared mechanistic axis when applied during early developmental windows. Perinatal supplementation with vitamins [136,137], NO precursors [138,139], and sulfur-containing amino acids [140] preserves nephron endowment, mitigates oxidative injury, and prevents the transition from prehypertension to overt disease. Importantly, these benefits are highly timing-dependent, reflecting intervention during critical periods when redox signaling exerts maximal influence on organogenesis and long-term cardiometabolic programming. These protective effects extend beyond direct redox modulation and involve enhancement of complementary signaling networks, including increased hydrogen sulfide bioavailability and gut microbiota remodeling.

Melatonin has emerged as a particularly potent reprogramming agent, combining free radical scavenging with a reduction in ADMA levels and upregulation of endogenous antioxidant defenses. Across multiple adverse developmental exposures, perinatal melatonin therapy consistently prevents programmed hypertension in rodent models [141,142]. Its efficacy underscores a shift from non-specific ROS neutralization toward modulation of endogenous redox-regulatory pathways. Polyphenols such as resveratrol further extend this paradigm by integrating antioxidant and prebiotic actions, restoring microbial diversity and limiting oxidative DNA damage in offspring exposed to various maternal insults [143,144]. Synthetic antioxidants provide additional mechanistic precision; N-acetylcysteine replenishes glutathione stores and rebalances NO–H_2_S signaling [145,146], while mitochondria-targeted compounds selectively protect against nephron loss and kidney injury in mouse models of maternal smoking-induced oxidative stress [147]. These targeted approaches emphasize pathway- and compartment-specific redox modulation rather than global ROS suppression, thereby preserving physiological redox signaling.

Despite robust preclinical efficacy, translation into clinical practice remains challenging [148,149]. While antioxidant therapy demonstrates protective effects in animal models of cardiovascular and CKMS-related injury, human trials—particularly those relying on chronic, non-specific antioxidant supplementation—often yield limited benefits due to species-specific differences in metabolism and redox regulation, heterogeneity in patient populations, variations in timing and dosing, and the presence of comorbidities or polypharmacy that are rarely modeled preclinically [150,151]. Furthermore, prolonged antioxidant use may blunt physiological ROS signaling, which is essential for vascular homeostasis, immune surveillance, and normal development. These observations reinforce the need to distinguish developmental reprogramming interventions from failed adult antioxidant paradigms and to adopt precise, temporally optimized, and mechanism-based strategies.

Optimal dosing, timing, and duration of antioxidant interventions have not been fully established, and excessive supplementation may induce “antioxidant stress,” potentially disrupting physiologic redox signaling essential for normal development. To date, more than 200 clinical trials have evaluated antioxidant use during pregnancy [152], yet most were not designed to assess long-term offspring cardiovascular–kidney–metabolic outcomes, underscoring the lack of definitive clinical proof-of-concept. Within this context, breastfeeding represents a physiologically aligned and safe reprogramming strategy, delivering a complex repertoire of antioxidants, bioactive molecules, and beneficial microbes that collectively enhance renal and cardiovascular resilience [153]. Collectively, these findings support antioxidant-based reprogramming as a promising but still experimental precision-prevention strategy, requiring rigorous translational validation before clinical implementation. Together, these data position precision prevention as an early, mechanism-based approach that leverages developmental plasticity to durably modify CKMS trajectories across the life course.

#### 6.3.2. Precision Nutrition

Precision nutrition constitutes a complementary pillar of precision prevention in CKMS. By moving beyond “one-size-fits-all” dietary recommendations, this paradigm accounts for interindividual variability in biological, environmental, and behavioral determinants of health. Targeted nutritional strategies are thereby deployed to preserve homeostasis, slow disease progression, and mitigate long-term cardiovascular and renal complications within the CKMS framework [154,155].

A cornerstone of precision nutrition is the use of multimetabolite signatures to objectively quantify dietary adherence and cardiometabolic risk. In the PREDIMED trial, a panel of 67 plasma metabolites substantially improved cardiovascular risk prediction. Elevated ceramides, acylcarnitines, and branched-chain amino acids were consistently associated with increased CVD risk [156,157]; importantly, adherence to a Mediterranean dietary pattern attenuated these adverse associations, indicating that individuals with high-risk metabotypes derive the greatest benefit from targeted lipid-quality interventions [158].

Landmark studies further support phenotype-guided dietary tailoring. The PERSON trial demonstrated that individuals with insulin resistance primarily driven by hepatic dysfunction respond more favorably to diets enriched in monounsaturated fats than to conventional low-fat, high-fiber regimens [159]. Advances in machine learning have expanded this precision framework by integrating gut microbiome profiles with clinical markers to predict postprandial glucose responses [160]. Algorithm-derived personalized diets outperform standardized carbohydrate counting in maintaining normoglycemia, thereby disrupting cycles of oxidative stress triggered by transient hyperglycemia.

Sustainable implementation of precision prevention requires integration of behavioral economics and digital health tools. The ChooseWell program demonstrated that individuals with a genetic predisposition for carbohydrate preference are particularly responsive to environmental nudges, such as traffic-light food labeling [161]. In parallel, the MINISTOP mobile health intervention illustrates the effectiveness of culturally adapted digital tools in pediatric populations, reducing sugary beverage intake and supporting healthier growth trajectories in underserved communities [162].

Collectively, evidence from PREDIMED, PERSON, and related trials underscores precision nutrition as an essential component of CKMS risk reduction. By shifting prevention from reactive management to proactive, data-driven intervention, precision nutrition enables more effective, personalized, and equitable strategies to address the intertwined cardiovascular, renal, and metabolic dimensions of CKMS.

### 6.4. Precision Treatment: Phenotype-Guided Pharmacotherapy

The therapeutic management of CKMS has progressed from uniform risk-factor control toward precision treatment grounded in dominant pathophysiologic phenotypes. This paradigm leverages next-generation cardiometabolic agents with pleiotropic, multi-organ protective effects that extend well beyond glycemic regulation, thereby enabling targeted, mechanism-based intervention across the CKMS continuum [2,163].

Precision treatment also represents the most advanced clinical application of precision medicine in pediatric obesity [108]. Genetically informed therapies, such as metreleptin for congenital leptin deficiency and setmelanotide for disorders involving POMC, PCSK1, LEPR, and Bardet–Biedl syndrome, illustrate how molecular diagnosis can directly inform effective pharmacologic intervention. These targeted treatments achieve clinically meaningful weight reduction while improving metabolic profiles and quality of life without compromising linear growth. Although pharmacogenomic guidance for commonly prescribed anti-obesity medications remains limited, these examples clearly demonstrate the feasibility and impact of genotype-driven precision therapy.

Sodium–glucose cotransporter-2 (SGLT2) inhibitors have emerged as the cornerstone of precision treatment for heart failure– and CKD–dominant phenotypes [164]. Through combined hemodynamic, anti-inflammatory, and metabolic effects—including a reduction in intraglomerular hypertension and improvement in myocardial energetics—SGLT2 inhibitors confer consistent clinical benefits independent of diabetes status. Landmark heart failure trials demonstrated relative risk reductions of approximately one quarter in cardiovascular mortality [165], while dedicated kidney outcome trials confirmed substantial and consistent reductions in major adverse renal and cardiovascular events, including among patients with advanced CKD [166]. Collectively, these data establish SGLT2 inhibitors as foundational therapy for stabilizing kidney function and delaying progression to renal replacement therapy.

In contrast, CKMS phenotypes driven primarily by excess adiposity or established atherosclerotic CVD are optimally addressed with incretin-based therapies. Glucagon-like peptide-1 receptor agonists and dual GIP/GLP-1 receptor agonists directly target the metabolic, inflammatory, and neurohormonal drivers of obesity and atherosclerosis [167]. High-dose semaglutide has demonstrated significant reductions in major adverse cardiovascular events among individuals with established ASCVD without diabetes, alongside marked and durable weight loss and improvement in cardiometabolic liver disease [168]. Tirzepatide further extends this therapeutic class by achieving unprecedented weight reduction exceeding 20% and significantly lowering the composite risk of cardiovascular death and heart-failure events in patients with obesity-related HFpEF, underscoring adiposity regression as a disease-modifying strategy [169].

For patients with diabetic kidney disease characterized by persistent albuminuria despite optimized renin–angiotensin system blockade, finerenone provides a precision-targeted anti-fibrotic approach [170]. As a selective non-steroidal mineralocorticoid receptor antagonist (MRA), finerenone attenuates inflammatory and fibrotic signaling within both renal and cardiovascular tissues. Large outcome trials have demonstrated meaningful reductions in kidney disease progression and cardiovascular events when finerenone is added to standard therapy [171], with particular benefit observed in patients who retain residual renal risk despite concurrent SGLT2 inhibitor treatment [172].

Accordingly, precision treatment in CKMS depends on aligning pharmacologic mechanisms with the dominant clinical driver: hemodynamic and renal stress are addressed through SGLT2 inhibition; metabolic overload and atherosclerotic risk through incretin-based therapies; and residual inflammatory–fibrotic injury through selective mineralocorticoid receptor antagonism. Emerging evidence supports integrated combination strategies for patients with confluent high-risk phenotypes, in whom concurrent use of SGLT2 inhibitors, GLP-1 receptor agonists, and MRAs may substantially extend event-free survival compared with conventional care. SGLT2 inhibitors improve redox balance by reducing mitochondrial and NADPH oxidase-derived ROS, enhancing endogenous antioxidant enzyme activity, and mitigating hyperglycemia-induced oxidative stress in renal and cardiovascular tissues [173,174]. GLP-1 receptor agonists exert antioxidant effects via activation of cAMP/PKA and AMPK signaling pathways, upregulating endogenous antioxidants such as superoxide dismutase and glutathione peroxidase, and limiting ROS-mediated endothelial and kidney injury [173,174]. These mechanistic insights extend beyond their systemic metabolic and hemodynamic benefits and emphasize their relevance to antioxidant and redox-targeted interventions. Notably, these therapeutic classes converge on pathways that reduce oxidative stress and restore redox homeostasis [175,176,177], highlighting their potential relevance to precision redox-based prevention strategies, including earlier life-course intervention.

However, prolonged antioxidant supplementation or chronic suppression of inflammatory or stress-responsive pathways may exert context-dependent effects and could theoretically attenuate oxidative or immune-mediated tumor surveillance, thereby favoring tumor survival in select settings [178,179]. These considerations highlight the importance of timing, dosage, and patient selection, and indicate that long-term safety—particularly with respect to cancer risk—warrants careful evaluation in future clinical studies.

## 7. Conclusions

Redox regulation during fetal and perinatal development plays a decisive role in shaping long-term cardiovascular, renal, and metabolic health, thereby modulating individual susceptibility to CKMS later in life. Despite growing recognition of these developmental origins, current clinical practice remains largely anchored in guideline-based, fragmented care, with limited capacity to address life-course risk stratification, early precision prevention through developmental reprogramming, or stabilization of interconnected organ networks [2,180].

Precision medicine offers an integrative framework to address these gaps by enabling refined multisystem phenotyping, risk prediction through the integration of genetic susceptibility, environmental exposures, and longitudinal profiles, and personalized prevention and treatment strategies tailored to individual biological and contextual factors. However, its application in CKMS is constrained by the syndrome’s multiorgan, dynamic nature, the context-dependent role of redox signaling, prolonged latency between early-life programming and overt disease, and the scarcity of validated biomarkers, longitudinal cohorts, and precision-guided interventions. Additional challenges related to data integration, cost, and equitable implementation further limit clinical translation.

The next phase of redox-driven precision medicine in CKMS must therefore operationalize life-course biology, transforming redox signaling from a mechanistic concept into a predictive, preventive, and personalized clinical framework. Progress will depend on accurate timing, biologically informed phenotyping, and systems-level integration rather than single-target or late-stage interventions.

## Data Availability

No new data were created or analyzed in this study. Data sharing is not applicable to this article.

## References

[1] Vaduganathan M., Mensah G.A., Turco J.V., Fuster V., Roth G.A. (2022). The global burden of cardiovascular diseases and risk: A compass for future health. J. Am. Coll. Cardiol..

[2] Ndumele C.E., Rangaswami J., Chow S.L., Neeland I.J., Tuttle K.R., Khan S.S., Coresh J., Mathew R.O., Baker-Smith C.M., Carnethon M.R. (2023). Cardiovascular-Kidney-Metabolic Health: A Presidential Advisory from the American Heart Association. Circulation.

[3] Aggarwal R., Ostrominski J.W., Vaduganathan M. (2024). Prevalence of Cardiovascular-Kidney-Metabolic Syndrome Stages in US Adults, 2011–2020. JAMA.

[4] Barker D.J. (2000). In utero programming of cardiovascular disease. Theriogenology.

[5] Hoffman D.J., Powell T.L., Barrett E.S., Hardy D.B. (2021). Developmental origins of metabolic diseases. Physiol. Rev..

[6] Iturzaeta A., Sáenz Tejeira M.M. (2022). Early programming of hypertension. Arch. Argent. Pediatr..

[7] Tain Y.L. (2025). Advocacy for DOHaD research optimizing child kidney health. Pediatr. Neonatol..

[8] Fleming T.P., Velazquez M.A., Eckert J.J. (2015). Embryos, DOHaD and David Barker. J. Dev. Orig. Health Dis..

[9] Tain Y.L., Joles J.A. (2015). Reprogramming: A Preventive Strategy in Hypertension Focusing on the Kidney. Int. J. Mol. Sci..

[10] Aljunaidy M.M., Morton J.S., Cooke C.M., Davidge S.T. (2017). Prenatal hypoxia and placental oxidative stress: Linkages to developmental origins of cardiovascular disease. Am. J. Physiol. Regul. Integr. Comp. Physiol..

[11] Pizzino G., Irrera N., Cucinotta M., Pallio G., Mannino F., Arcoraci V., Squadrito F., Altavilla D., Bitto A. (2017). Oxidative Stress: Harms and Benefits for Human Health. Oxidative Med. Cell. Longev..

[12] Tain Y.L., Lin Y.J., Hsu C.N. (2025). Animal Models for Studying Developmental Origins of Cardiovascular-Kidney-Metabolic Syndrome. Biomedicines.

[13] Lu P.C., Tain Y.L., Lin Y.J., Hsu C.N. (2025). Oxidative Stress in Maternal and Offspring Kidney Disease and Hypertension: A Life-Course Perspective. Antioxidants.

[14] Rodríguez-Rodríguez P., Ramiro-Cortijo D., Reyes-Hernández C.G., López de Pablo A.L., González M.C., Arribas S.M. (2018). Implication of Oxidative Stress in Fetal Programming of Cardiovascular Disease. Front. Physiol..

[15] Tain Y.L., Hsu C.N. (2022). Metabolic Syndrome Programming and Reprogramming: Mechanistic Aspects of Oxidative Stress. Antioxidants.

[16] Wilcox C.S. (2005). Oxidative stress and nitric oxide deficiency in the kidney: A critical link to hypertension?. Am. J. Physiol. Regul. Integr. Comp. Physiol..

[17] Daenen K., Andries A., Mekahli D., Van Schepdael A., Jouret F., Bammens B. (2019). Oxidative stress in chronic kidney disease. Pediatr. Nephrol..

[18] Förstermann U. (2006). Janus-faced role of endothelial NO synthase in vascular disease: Uncoupling of oxygen reduction from NO synthesis and its pharmacological reversal. Biol. Chem..

[19] Calabrese V., Cornelius C., Rizzarelli E., Owen J.B., Dinkova-Kostova A.T., Butterfield D.A. (2009). Nitric oxide in cell survival: A janus molecule. Antioxid. Redox Signal..

[20] Vornic I., Buciu V., Furau C.G., Gaje P.N., Ceausu R.A., Dumitru C.S., Barb A.C., Novacescu D., Cumpanas A.A., Latcu S.C. (2024). Oxidative Stress and Placental Pathogenesis: A Contemporary Overview of Potential Biomarkers and Emerging Therapeutics. Int. J. Mol. Sci..

[21] Pohlman N., Patel P.N., Essien U.R., Tang J.J., Joseph J.J. (2025). Novel Cardiometabolic Medications in the Cardiovascular-Kidney-Metabolic Syndrome Era. J. Clin. Endocrinol. Metab..

[22] Xu X., Shao X., Hou F.F. (2025). Risk stratification of metabolic disorder-associated kidney disease. Kidney Int..

[23] Spahis S., Borys J.M., Levy E. (2017). Metabolic Syndrome as a Multifaceted Risk Factor for Oxidative Stress. Antioxid. Redox Signal..

[24] Ravarotto V., Simioni F., Pagnin E., Davis P.A., Calò L.A. (2018). Oxidative stress-chronic kidney disease-cardiovascular disease: A vicious circle. Life Sci..

[25] Quaggin S.E., Magod B. (2024). A united vision for cardiovascular-kidney-metabolic health. Nat. Rev. Nephrol..

[26] Khan S.S., Coresh J., Pencina M.J., Ndumele C.E., Rangaswami J., Chow S.L., Palaniappan L.P., Sperling L.S., Virani S.S., Ho J.E. (2023). Novel Prediction Equations for Absolute Risk Assessment of Total Cardiovascular Disease Incorporating Cardiovascular-Kidney-Metabolic Health: A Scientific Statement From the American Heart Association. Circulation.

[27] Afkarian M., Sachs M.C., Kestenbaum B., Hirsch I.B., Tuttle K.R., Himmelfarb J., de Boer I.H. (2013). Kidney disease and increased mortality risk in type 2 diabetes. J. Am. Soc. Nephrol..

[28] He Q., Sun M., Wang Q., Huang J., Hu S., Shen Y., Li L. (2026). Cardiovascular-kidney-metabolic syndrome, dementia risk, cognition, and neuroimaging outcomes. J. Affect. Disord..

[29] Fleming T.P., Watkins A.J., Velazquez M.A., Mathers J.C., Prentice A.M., Stephenson J., Barker M., Saffery R., Yajnik C.S., Eckert J.J. (2018). Origins of lifetime health around the time of conception: Causes and consequences. Lancet.

[30] Palaniappan L., Wong E., Shin J., Fortmann S.P., Lauderdale D.S. (2011). Asian Americans have greater prevalence of metabolic syndrome despite lower body mass index. Int. J. Obes..

[31] Larque E., Labayen I., Flodmark C., Lissau I., Czernin S., Moreno L.A., Pietrobelli A., Widhalm K. (2019). From conception to infancy: Early risk factors for childhood obesity. Nat. Rev. Endocrinol..

[32] Page K.A., Luo S., Wang X., Chow T., Alves J., Buchanan T.A., Xiang A.H. (2019). Children exposed to maternal obesity or gestational diabetes mellitus during early fetal development have hypothalamic alterations that predict future weight gain. Diabetes Care.

[33] Tain Y.L., Hsu C.N. (2022). Cardiovascular Risks of Hypertension: Lessons from Children with Chronic Kidney Disease. Children.

[34] Giugni F.R., Berry J.D., Khera A., Shah A.M., de Lemos J.A. (2024). Precision Medicine for Cardiovascular Prevention and Population Health: A Bridge Too Far?. Circulation.

[35] Thangaraj P.M., Benson S.H., Oikonomou E.K., Asselbergs F.W., Khera R. (2024). Cardiovascular care with digital twin technology in the era of generative artificial intelligence. Eur. Heart J..

[36] Franks P.W., Cefalu W.T., Dennis J., Florez J.C., Mathieu C., Morton R.W., Ridderstråle M., Sillesen H.H., Stehouwer C.D.A. (2023). Precision medicine for cardiometabolic disease: A framework for clinical translation. Lancet Diabetes Endocrinol..

[37] Thompson L.P., Al-Hasan Y. (2012). Impact of oxidative stress in fetal programming. J. Pregnancy.

[38] Grzeszczak K., Łanocha-Arendarczyk N., Malinowski W., Ziętek P., Kosik-Bogacka D. (2023). Oxidative Stress in Pregnancy. Biomolecules.

[39] Carter A.M. (2000). Placental oxygen consumption. Part I. In vivo studies—A review. Placenta.

[40] Dennery P.A. (2010). Oxidative stress in development: Nature or nurture?. Free Radic. Biol. Med..

[41] Tain Y.L., Lee W.C., Wu K.L.H., Leu S., Chan J.Y.H. (2016). Targeting arachidonic acid pathway to prevent programmed hypertension in maternal fructose-fed male adult rat offspring. J. Nutr. Biochem..

[42] Ito J., Nakagawa K., Kato S., Miyazawa T., Kimura F., Miyazawa T. (2016). The combination of maternal and offspring high-fat diets causes marked oxidative stress and development of metabolic syndrome in mouse offspring. Life Sci..

[43] Martínez Gascón L.E., Ortiz M.C., Galindo M., Sanchez J.M., Sancho-Rodriguez N., Albaladejo Otón M.D., Rodriguez Mulero M.D., Rodriguez F. (2022). Role of heme oxygenase in the regulation of the renal hemodynamics in a model of sex dependent programmed hypertension by maternal diabetes. Am. J. Physiol.-Regul. Integr. Comp. Physiol..

[44] Hsu C.N., Yang H.W., Hou C.Y., Chang-Chien G.P., Lin S., Tain Y.L. (2020). Maternal Adenine-Induced Chronic Kidney Disease Programs Hypertension in Adult Male Rat Offspring: Implications of Nitric Oxide and Gut Microbiome Derived Metabolites. Int. J. Mol. Sci..

[45] Ojeda N.B., Hennington B.S., Williamson D.T., Hill M.L., Betson N.E., Sartori-Valinotti J.C., Reckelhoff J.F., Royals T.P., Alexander B.T. (2012). Oxidative stress contributes to sex differences in blood pressure in adult growth-restricted offspring. Hypertension.

[46] Vargas V.E., Gurung S., Grant B., Hyatt K., Singleton K., Myers S.M., Saunders D., Njoku C., Towner R., Myers D.A. (2017). Gestational hypoxia disrupts the neonatal leptin surge and programs hyperphagia and obesity in male offspring in the Sprague-Dawley rat. PLoS ONE.

[47] Tain Y.L., Sheen J.M., Chen C.C., Yu H.R., Tiao M.M., Kuo H.C., Huang L.T. (2014). Maternal citrulline supplementation prevents prenatal dexamethasone-induced programmed hypertension. Free Radic. Res..

[48] Hsu C.N., Tain Y.L. (2021). Adverse Impact of Environmental Chemicals on Developmental Origins of Kidney Disease and Hypertension. Front. Endocrinol..

[49] Wei Z., Song L., Wei J., Chen T., Chen J., Lin Y., Xia W., Xu B., Li X., Chen X. (2012). Maternal Exposure to Di-(2-Ethylhexyl) Phthalate Alters Kidney Development Through the Renin-Angiotensin System in Offspring. Toxicol. Lett..

[50] Tain Y.L., Hsu C.N. (2023). The NOS/NO System in Renal Programming and Reprogramming. Antioxidants.

[51] Tsikas D. (2017). Does the inhibitory action of asymmetric dimethylarginine (ADMA) on the endothelial nitric oxide synthase activity explain its importance in the cardiovascular system? The ADMA paradox. J. Controv. Biomed. Res..

[52] Teerlink T., Luo Z., Palm F., Wilcox C.S. (2009). Cellular ADMA: Regulation and action. Pharmacol. Res..

[53] Divvela S.S.K., Gallorini M., Gellisch M., Patel G.D., Saso L., Brand-Saberi B. (2025). Navigating redox imbalance: The role of oxidative stress in embryonic development and long-term health outcomes. Front. Cell Dev. Biol..

[54] Cuadrado A., Rojo A.I., Wells G., Hayes J.D., Cousin S.P., Rumsey W.L., Attucks O.C., Franklin S., Levonen A.L., Kensler T.W. (2019). Therapeutic targeting of the NRF2 and KEAP1 partnership in chronic diseases. Nat. Rev. Drug Discov..

[55] Kračun D., Lopes L.R., Cifuentes-Pagano E., Pagano P.J. (2025). NADPH oxidases: Redox regulation of cell homeostasis and disease. Physiol. Rev..

[56] Spinelli J.B., Haigis M.C. (2018). The multifaceted contributions of mitochondria to cellular metabolism. Nat. Cell Biol..

[57] Piacenza L., Zeida A., Trujillo M., Radi R. (2022). The superoxide radical switch in the biology of nitric oxide and peroxynitrite. Physiol. Rev..

[58] Kett M.M., Denton K.M. (2011). Renal programming: Cause for concern?. Am. J. Physiol. Regul. Integr. Comp. Physiol..

[59] Tain Y.L., Hsieh C.S., Lin I.C., Chen C.C., Sheen J.M., Huang L.T. (2010). Effects of maternal L-citrulline supplementation on renal function and blood pressure in offspring exposed to maternal caloric restriction: The impact of nitric oxide pathway. NitricOxide.

[60] Cambonie G., Comte B., Yzydorczyk C., Ntimbane T., Germain N., Lê N.L., Pladys P., Gauthier C., Lahaie I., Abran D. (2007). Antenatal antioxidant prevents adult hypertension, vascular dysfunction, and microvascular rarefaction associated with in utero exposure to a low-protein diet. Am. J. Physiol. Regul. Integr. Comp. Physiol..

[61] Tain Y.L., Chen C.C., Sheen J.M., Yu H.R., Tiao M.M., Kuo H.C., Huang L.T. (2014). Melatonin attenuates prenatal dexamethasone-induced blood pressure increase in a rat model. J. Am. Soc. Hypertens..

[62] Stangenberg S., Nguyen L.T., Chen H., Al-Odat I., Killingsworth M.C., Gosnell M.E., Anwer A.G., Goldys E.M., Pollock C.A., Saad S. (2015). Oxidative stress, mitochondrial perturbations and fetal programming of renal disease induced by maternal smoking. Int. J. Biochem. Cell Biol..

[63] Bertram J.F., Douglas-Denton R.N., Diouf B., Hughson M.D., Hoy W.E. (2011). Human nephron number: Implications for health and disease. Pediatr. Nephrol..

[64] Nenov V.D., Taal M.W., Sakharova O.V., Brenner B.M. (2000). Multi-hit nature of chronic renal disease. Curr. Opin. Nephrol. Hypertens..

[65] Rana M.N., Neeland I.J. (2022). Adipose tissue inflammation and cardio-vascular disease: An update. Curr. Diab. Rep..

[66] Neeland I.J., Ross R., Després J.P., Matsuzawa Y., Yamashita S., Shai I., Seidell J., Magni P., Santos R.D., Arsenault B. (2019). Visceral and ectopic fat, atherosclerosis, and cardiometabolic disease: A position statement. Lancet Diabetes Endocrinol..

[67] Halim M., Halim A. (2019). The effects of inflammation, aging and oxidative stress on the pathogenesis of diabetes mellitus (type 2 diabetes). Diabetes Metab. Syndr..

[68] Blackmore H.L., Ozanne S.E. (2015). Programming of cardiovascular disease across the life-course. J. Mol. Cell. Cardiol..

[69] Brain K.L., Allison B.J., Niu Y., Cross C.M., Itani N., Kane A.D., Herrera E.A., Skeffington K.L., Botting K.J., Giussani D.A. (2019). Intervention against hypertension in the next generation programmed by developmental hypoxia. PLoS Biol..

[70] Vieira L.D., Farias J.S., de Queiroz D.B., Cabral E.V., Lima-Filho M.M., Sant’Helena B.R.M., Aires R.S., Ribeiro V.S., Santos Rocha J., Xavier F.E. (2018). Oxidative stress induced by prenatal LPS leads to endothelial dysfunction and renal haemodynamic changes through angiotensin II/NADPH oxidase pathway: Prevention by early treatment with α-tocopherol. Biochim. Biophys. Acta Mol. Basis Dis..

[71] Thornburg K.L. (2015). The programming of cardiovascular disease. J. Dev. Orig. Health Dis..

[72] Giam B., Kaye D.M., Rajapakse N.W. (2016). Role of Renal Oxidative Stress in the Pathogenesis of the Cardiorenal Syndrome. Heart Lung Circ..

[73] Hsu C.N., Tain Y.L. (2020). Early Origins of Hypertension: Should Prevention Start Before Birth Using Natural Antioxidants?. Antioxidants.

[74] Hsu C.N., Lin Y.J., Hou C.Y., Chen Y.W., Tain Y.L. (2025). Early-Life Prevention of Cardiovascular-Kidney-Metabolic Syndrome: The DOHaD Perspective on Resveratrol and Short-Chain Fatty Acids. Antioxidants.

[75] Sladek S.M., Magness R.R., Conrad K.P. (1997). Nitric oxide and pregnancy. Am. J. Physiol..

[76] Huang L.T., Hsieh C.S., Chang K.A., Tain Y.L. (2012). Roles of nitric oxide and asymmetric dimethylarginine in pregnancy and fetal programming. Int. J. Mol. Sci..

[77] Tain Y.L., Lee W.C., Hsu C.N., Lee W.C., Huang L.T., Lee C.T., Lin C.Y. (2013). Asymmetric dimethylarginine is associated with developmental programming of adult kidney disease and hypertension in offspring of streptozotocin-treated mothers. PLoS ONE.

[78] Pettersson A., Hedner T., Milsom I. (1998). Increased circulating concentrations of asymmetric dimethyl arginine (ADMA), an endogenous inhibitor of nitric oxide synthesis, in preeclampsia. Acta Obstet. Gynecol. Scand..

[79] Akturk M., Altinova A., Mert I., Dincel A., Sargin A., Buyukkagnici U., Arslan M., Danisman N. (2010). Asymmetric dimethylarginine concentrations are elevated in women with gestational diabetes. Endocrine.

[80] Basu S. (2008). F2-isoprostanes in human health and diseases: From molecular mechanisms to clinical implications. Antioxid. Redox Signal..

[81] Longini M., Perrone S., Kenanidis A., Vezzosi P., Marzocchi B., Petraglia F., Centini G., Buonocore G. (2005). Isoprostanes in amniotic fluid: A predictive marker for fetal growth restriction in pregnancy. Free Radic. Biol. Med..

[82] Graille M., Wild P., Sauvain J.J., Hemmendinger M., Guseva Canu I., Hopf N.B. (2020). Urinary 8-OHdG as a Biomarker for Oxidative Stress: A Systematic Literature Review and Meta-Analysis. Int. J. Mol. Sci..

[83] Do Nascimento L.C.P., Neto J.P.R.C., de Andrade Braga V., Lagranha C.J., de Brito Alves J.L. (2020). Maternal exposure to high-fat and high-cholesterol diet induces arterial hypertension and oxidative stress along the gut-kidney axis in rat offspring. Life Sci..

[84] Saad A.F., Dickerson J., Kechichian T.B., Yin H., Gamble P., Salazar A., Patrikeev I., Motamedi M., Saade G.R., Costantine M.M. (2016). High-fructose diet in pregnancy leads to fetal programming of hypertension, insulin resistance, and obesity in adult offspring. Am. J. Obstet. Gynecol..

[85] Ciesielska S., Slezak-Prochazka I., Bil P., Rzeszowska-Wolny J. (2021). Micro RNAs in Regulation of Cellular Redox Homeostasis. Int. J. Mol. Sci..

[86] Bian X., Liu J., Yang Q., Liu Y., Jia W., Zhang X., Li Y.X., Shao X., Wang Y.L. (2021). MicroRNA-210 regulates placental adaptation to maternal hypoxic stress during pregnancy. Biol. Reprod..

[87] Okamura D.M., Himmelfarb J. (2009). Tipping the redox balance of oxidative stress in fibrogenic pathways in chronic kidney disease. Pediatr. Nephrol..

[88] Drożdż D., Kwinta P., Sztefko K., Kordon Z., Drożdż T., Łątka M., Miklaszewska M., Zachwieja K., Rudzin′ski A., Pietrzyk J.A. (2016). Oxidative Stress Biomarkers and Left Ventricular Hypertrophy in Children with Chronic Kidney Disease. Oxidative Med. Cell. Longev..

[89] Kishi S., Nagasu H., Kidokoro K., Kashihara N. (2024). Oxidative stress and the role of redox signalling in chronic kidney disease. Nat. Rev. Nephrol..

[90] Mitsnefes M.M. (2021). Cardiovascular Disease Risk Factors in Chronic Kidney Disease in Children. Semin. Nephrol..

[91] Velasquez M.T., Ramezani A., Manal A., Raj D.S. (2016). Trimethylamine N-Oxide: The good, the bad and the unknown. Toxins.

[92] Dalpathadu H., Salim A.M., Wade A., Greenway S.C. (2024). A Systematic Review of Uremic Toxin Concentrations and Cardiovascular Risk Markers in Pediatric Chronic Kidney Disease. Toxins.

[93] Schena F.P., Cox S.N. (2018). Biomarkers and Precision Medicine in IgA Nephropathy. Semin. Nephrol..

[94] Li W., Shen C., Kong W., Zhou X., Fan H., Zhang Y., Liu Z., Zheng L. (2024). Association between the triglyceride glucose-body mass index and future cardiovascular disease risk in a population with Cardiovascular-Kidney-Metabolic syndrome stage 0–3: A nationwide prospective cohort study. Cardiovasc. Diabetol..

[95] Zheng Q., Cao Z., Teng J., Lu Q., Huang P., Zhou J. (2025). Association between atherogenic index of plasma with all-cause and cardiovascular mortality in individuals with Cardiovascular-Kidney-Metabolic syndrome. Cardiovasc. Diabetol..

[96] Shahraki N., Samadi S., Arasteh O., Dashtbayaz R.J., Zarei B., Mohammadpour A.H., Jomehzadeh V. (2024). Cardiac troponins and coronary artery calcium score: A systematic review. BMC Cardiovasc. Disord..

[97] Castiglione V., Aimo A., Vergaro G., Saccaro L., Passino C., Emdin M. (2022). Biomarkers for the diagnosis and management of heart failure. Heart Fail. Rev..

[98] (2024). Kidney Disease: Improving Global Outcomes (KDIGO) CKD Work Group. KDIGO 2024 Clinical Practice Guideline for the Evaluation and Management of Chronic Kidney Disease. Kidney Int..

[99] Claudel S.E., Verma A. (2025). Albuminuria in Cardiovascular, Kidney, and Metabolic Disorders: A State-of-the-Art Review. Circulation.

[100] Medić B., Rovčanin B., Basta Jovanović G., Radojević-Škodrić S., Prostran M. (2015). Kidney Injury Molecule-1 and Cardiovascular Diseases: From Basic Science to Clinical Practice. BioMed Res. Int..

[101] Stopic B., Medic-Brkic B., Savic-Vujovic K., Davidovic Z., Todorovic J., Dimkovic N. (2022). Biomarkers and Predictors of Adverse Cardiovascular Events in Different Stages of Chronic Kidney Disease. Dose Response.

[102] Moreira J.M., da Silva A.N., Marciano Vieira É.L., Teixeira A.L., Kummer A.M., Simões E., Silva A.C. (2019). Soluble tumor necrosis factor receptors are associated with severity of kidney dysfunction in pediatric chronic kidney disease. Pediatr. Nephrol..

[103] Rossaint J., Unruh M., Zarbock A. (2017). Fibroblast growth factor 23 actions in inflammation: A key factor in CKD outcomes. Nephrol. Dial. Transplant..

[104] Hanna M.H., Dalla Gassa A., Mayer G., Zaza G., Brophy P.D., Gesualdo L., Pesce F. (2017). The nephrologist of tomorrow: Towards a kidney-omic future. Pediatr. Nephrol..

[105] Martínez-González M.A., Planes F.J., Ruiz-Canela M., Toledo E., Estruch R., Salas-Salvadó J., Valdés-Más R., Mena P., Castañer O., Fitó M. (2025). Recent advances in precision nutrition and cardiometabolic diseases. Rev. Esp. Cardiol..

[106] Luévano-Contreras C., Gómez-Ojeda A., Macías-Cervantes M.H., Garay-Sevilla M.E. (2017). Dietary Advanced Glycation End Products and Cardiometabolic Risk. Curr. Diab. Rep..

[107] Moreno J.A., Hamza E., Guerrero-Hue M., Rayego-Mateos S., García-Caballero C., Vallejo-Mudarra M., Metzinger L., Metzinger-Le Meuth V. (2021). Non-Coding RNAs in Kidney Diseases: The Long and Short of Them. Int. J. Mol. Sci..

[108] Tillman E.M., Mertami S. (2024). Precision medicine to identify, prevent, and treat pediatric obesity. Pharmacotherapy.

[109] Silventoinen K., Rokholm B., Kaprio J., Sorensen T.I. (2010). The genetic and environmental influences on childhood obesity: A systematic review of twin and adoption studies. Int. J. Obes..

[110] Reznichenko A., Nair V., Eddy S., Fermin D., Tomilo M., Slidel T., Ju W., Henry I., Badal S.S., Wesley J.D. (2024). Unbiased kidney-centric molecular categorization of chronic kidney disease as a step towards precision medicine. Kidney Int..

[111] Ajoolabady A., Pratico D., Dunn W.B., Lip G.Y.H., Ren J. (2024). Metabolomics: Implication in cardiovascular research and diseases. Obes. Rev..

[112] Zhang K., Zhou H.Y., Baptista-Hon D.T., Gao Y., Liu X., Oermann E., Xu S., Jin S., Zhang J., Sun Z. (2024). International Consortium of Digital Twins in Medicine. Concepts and applications of digital twins in healthcare and medicine. Patterns.

[113] Shao X., Hu Y., Jia H., Song J. (2025). Digital Therapeutics in Cardiovascular Healthcare: A Narrative Review. Curr. Cardiol. Rep..

[114] Sabanayagam C., Banu R., Lim C., Tham Y.C., Cheng C.Y., Tan G., Ekinci E., Sheng B., McKay G., Shaw J.E. (2025). Artificial intelligence in chronic kidney disease management: A scoping review. Theranostics.

[115] Seyedi S.A., González-Rivas J.P., Mellacheruvu P., Mellacheruvu A., Aledavood S.P., Esteghamati A., Mechanick J.I. (2025). Cardiometabolic risk reduction with digital twinning in patients with type 2 diabetes. Cardiovasc. Diabetol. Endocrinol. Rep..

[116] Murugapoopathy V., Gupta I.R. (2020). A primer on congenital anomalies of the kidneys and urinary tracts (CAKUT). Clin. J. Am. Soc. Nephrol..

[117] Tain Y.L., Hsu C.N. (2024). Preterm Birth and Kidney Health: From the Womb to the Rest of Life. Children.

[118] Liu C., He Y., Venn A.J., Jose M.D., Tian J. (2023). Childhood modifiable risk factors and later life chronic kidney disease: A systematic review. BMC Nephrol..

[119] Yamagata K., Iseki K., Nitta K., Imai H., Iino Y., Matsuo S., Makino H., Hishida A. (2008). Chronic kidney disease perspectives in Japan and the importance of urinalysis screening. Clin. Exp. Nephrol..

[120] Lin C.Y., Sheng C.C., Lin C.C., Chen C.H., Chou P. (2001). Mass urinary screening and follow-up for school children in Taiwan Province. Acta Paediatr. Taiwanica.

[121] Yap H.K., Quek C.M., Shen Q., Joshi V., Chia K.S. (2005). Role of urinary screening programmes in children in the prevention of chronic kidney disease. Ann. Acad. Med..

[122] Hogg R.J. (2009). Screening for CKD in children: A global controversy. Clin. J. Am. Soc. Nephrol..

[123] Pottel H., Björk J., Courbebaisse M., Couzi L., Ebert N., Eriksen B.O., Dalton R.N., Dubourg L., Gaillard F., Garrouste C. (2021). Development and validation of a modified full age spectrum creatinine-based equation to estimate glomerular filtration rate: A cross-sectional analysis of pooled data. Ann. Intern. Med..

[124] Patel S.S., Daniels S.R. (2019). Ambulatory Blood Pressure Monitoring in Pediatrics. Curr. Hypertens. Rep..

[125] Htay H., Alrukhaimi M., Ashuntantang G.E., Bello A.K., Bellorin-Font E., Gharbi M.B., Braam B., Feehally J., Harris D.C., Jha V. (2018). Global access of patients with kidney disease to health technologies and medications: Findings from the Global Kidney Health Atlas project. Kidney Int. Suppl..

[126] Li X., Zhao W., Zhao L., Sun T., Pan H., Wang D. (2025). Cardiovascular disease risk estimates for primary prevention in the US prediabetes and diabetes population using the PREVENT equation. Diabetes Obes. Metab..

[127] Walther C.P., Gregg L.P., Navaneethan S.D. (2025). Cardiovascular Disease Risk Estimates in the US CKD Population Using the PREVENT Equation. Am. J. Kidney Dis..

[128] Vyas N., Zaheer A., Wijeysundera H.C. (2024). Untangling the Complex Multidimensionality of the Social Determinants of Cardiovascular Health: A Systematic Review. Can. J. Cardiol..

[129] Strocchi M., Hammersley D.J., Halliday B.P., Prasad S.K., Niederer S.A. (2025). Cardiac digital twins: A tool to investigate the function and treatment of the diabetic heart. Cardiovasc. Diabetol..

[130] Ward M., Yeganegi A., Baicu C.F., Bradshaw A.D., Spinale F.G., Zile M.R., Richardson W.J. (2022). Ensemble machine learning model identifies patients with HFpEF from matrix-related plasma biomarkers. Am. J. Physiol. Heart Circ. Physiol..

[131] Subramanian I., Verma S., Kumar S., Jere A., Anamika K. (2020). Multi-omics Data Integration, Interpretation, and Its Application. Bioinform. Biol. Insights.

[132] Neri L., Zhang H., Usvyat L.A. (2026). Artificial intelligence in kidney disease and dialysis: From data mining to clinical impact. Curr. Opin. Nephrol. Hypertens..

[133] Khan S.N., Danishuddin, Khan M.W.A., Guarnera L., Akhtar S.M.F. (2026). Multi-modal AI in precision medicine: Integrating genomics, imaging, and EHR data for clinical insights. Front. Artif. Intell..

[134] Frattolillo V., Massa A., Capone D., Monaco N., Forcina G., Di Filippo P., Marzuillo P., Miraglia Del Giudice E., Di Sessa A. (2025). Integrating digital health into pediatric obesity management: Current practices and future perspectives. Obes. Pillars.

[135] Hsu C.N., Tain Y.L. (2020). Developmental Origins of Kidney Disease: Why Oxidative Stress Matters?. Antioxidants.

[136] do Carmo Franco M., Ponzio B.F., Gomes G.N., Gil F.Z., Tostes R., Carvalho M.H., Fortes Z.B. (2009). Micronutrient prenatal supplementation prevents the development of hypertension and vascular endothelial damage induced by intrauterine malnutrition. Life Sci..

[137] Wang J., Yin N., Deng Y., Wei Y., Huang Y., Pu X., Li L., Zheng Y., Guo J., Yu J. (2016). Ascorbic Acid Protects against HypertensionthroughDownregulationofACE1Gene Expression Mediated by Histone Deacetylation in Prenatal InflammationInduced Offspring. Sci. Rep..

[138] Hsu C.N., Tain Y.L. (2019). Impact of Arginine Nutrition and Metabolism during Pregnancy on Offspring Outcomes. Nutrients.

[139] Chien S.J., Lin K.M., Kuo H.C., Huang C.F., Lin Y.J., Huang L.T., Tain Y.L. (2014). Two different approaches to restore renal nitric oxide and prevent hypertension in young spontaneously hypertensive rats: L-citrulline and nitrate. Transl. Res..

[140] Hsu C.N., Lin Y.J., Hou C.Y., Chen Y.W., Tain Y.L. (2025). Early-Life Hydrogen Sulfide Signaling as a Target for Cardiovascular-Kidney-Metabolic Syndrome Reprogramming. Antioxidants.

[141] Tain Y.L., Huang L.T., Hsu C.N. (2017). Developmental Programming of Adult Disease: Reprogramming by Melatonin?. Int. J. Mol. Sci..

[142] Tain Y.L., Hsu C.N. (2024). Melatonin Use during Pregnancy and Lactation Complicated by Oxidative Stress: Focus on Offspring’s Cardiovascular-Kidney-Metabolic Health in Animal Models. Antioxidants.

[143] Tain Y.L., Hsu C.N. (2024). Maternal Polyphenols and Offspring Cardiovascular-Kidney-Metabolic Health. Nutrients.

[144] Hsu C.N., Hou C.Y., Tain Y.L. (2021). Preventive Aspects of Early Resveratrol Supplementation in Cardiovascular and Kidney Disease of Developmental Origins. Int. J. Mol. Sci..

[145] Salamon S., Kramar B., Marolt T.P., Poljšak B., Milisav I. (2019). Medical and Dietary Uses of N-Acetylcysteine. Antioxidants.

[146] Tai I.H., Sheen J.M., Lin Y.J., Yu H.R., Tiao M.M., Chen C.C., Huang L.T., Tain Y.L. (2016). Maternal N-acetylcysteine therapy regulates hydrogen sulfide-generating pathway and prevents programmed hypertension in male offspring exposed to prenatal dexamethasone and postnatal high-fat diet. Nitric Oxide.

[147] Sukjamnong S., Chan Y.L., Zakarya R., Nguyen L.T., Anwer A.G., Zaky A.A., Santiyanont R., Oliver B.G., Goldys E., Pollock C.A. (2018). MitoQ supplementation prevent long-term impact of maternal smoking on renal development, oxidative stress and mitochondrial density in male mice offspring. Sci. Rep..

[148] Darby J.R.T., Mohd Dollah M.H.B., Regnault T.R.H., Williams M.T., Morrison J.L. (2019). Systematic review: Impact of resveratrol exposure during pregnancy on maternal and fetal outcomes in animal models of human pregnancy complications-Are we ready for the clinic?. Pharmacol. Res..

[149] Rosa A.C., Corsi D., Cavi N., Bruni N., Dosio F. (2021). Superoxide Dismutase Administration: A Review of Proposed Human Uses. Molecules.

[150] Nandha S.R., Checker R., Patwardhan R.S., Sharma D., Sandur S.K. (2025). Anti-oxidants as therapeutic agents for oxidative stress associated pathologies: Future challenges and opportunities. Free Radic. Res..

[151] Meulmeester F.L., Luo J., Martens L.G., Mills K., van Heemst D., Noordam R. (2022). Antioxidant Supplementation in Oxidative Stress-Related Diseases: What Have We Learned from Studies on Alpha-Tocopherol?. Antioxidants.

[152] (2025). ClinicalTrials.Gov. https://clinicaltrials.gov/.

[153] Tain Y.L., Lin Y.J., Hsu C.N. (2025). Breastfeeding and Future Cardiovascular, Kidney, and Metabolic Health—A Narrative Review. Nutrients.

[154] Mozaffarian D. (2016). Dietary and Policy Priorities for Cardiovascular Disease, Diabetes, and Obesity: A Comprehensive Review. Circulation.

[155] Guasch-Ferré M., Wittenbecher C., Palmnäs M., Ben-Yacov O., Blaak E.E., Dahm C.C., Fall T., Heitmann B.L., Licht T.R., Löf M. (2025). Precision nutrition for cardiometabolic diseases. Nat. Med..

[156] Wang D.D., Toledo E., Hruby A., Rosner B.A., Willett W.C., Sun Q., Razquin C., Zheng Y., Ruiz-Canela M., Guasch-Ferré M. (2017). Plasma Ceramides, Mediterranean Diet, and Incident Cardiovascular Disease in the PREDIMED Trial (Prevención con Dieta Mediterránea). Circulation.

[157] Li J., Guasch-Ferré M., Chung W., Ruiz-Canela M., Toledo E., Corella D., Bhupathiraju S.N., Tobias D.K., Tabung F.K., Hu J. (2020). The Mediterranean diet, plasma metabolome, and cardiovascular disease risk. Eur. Heart J..

[158] Konieczna J., Ruiz-Canela M., Galmes-Panades A.M., Abete I., Babio N., Fiol M., Martín-Sánchez V., Estruch R., Vidal J., Buil-Cosiales P. (2023). An Energy-Reduced Mediterranean Diet, Physical Activity, and Body Composition: An Interim Subgroup Analysis of the PREDIMED-Plus Randomized Clinical Trial. JAMA Netw. Open.

[159] Trouwborst I., Gijbels A., Jardon K.M., Siebelink E., Hul G.B., Wanders L., Erdos B., Péter S., Singh-Povel C.M., de Vogel-van den Bosch J. (2023). Cardiometabolic health improvements upon dietary intervention are driven by tissue-specific insulin resistance phenotype: A precision nutrition trial. Cell Metab..

[160] Bermingham K.M., Linenberg I., Polidori L., Asnicar F., Arrè A., Wolf J., Badri F., Bernard H., Capdevila J., Bulsiewicz W.J. (2024). Effects of a personalized nutrition program on cardiometabolic health: A randomized controlled trial. Nat. Med..

[161] Merino J., Dashti H.S., Levy D.E., Del Rocío Sevilla-González M., Hivert M.F., Porneala B.C., Saxena R., Thorndike A.N. (2023). Genetic predisposition to macronutrient preference and workplace food choices. Mol. Psychiatry.

[162] Alexandrou C., Henriksson H., Henström M., Henriksson P., Delisle Nyström C., Bendtsen M., Löf M. (2023). Effectiveness of a Smartphone App (MINISTOP 2.0) integrated in primary child health care to promote healthy diet and physical activity behaviors and prevent obesity in preschool-aged children: Randomized controlled trial. Int. J. Behav. Nutr. Phys. Act..

[163] Ferdinand K.C. (2024). An overview of cardiovascular-kidney-metabolic syndrome. Am. J. Manag. Care.

[164] Patel S.M., Kang Y.M., Im K., Neuen B.L., Anker S.D., Bhatt D.L., Butler J., Cherney D.Z.I., Claggett B.L., Fletcher R.A. (2024). Sodium-Glucose Cotransporter-2 Inhibitors and Major Adverse Cardiovascular Outcomes: A SMART-C Collaborative Meta-Analysis. Circulation.

[165] McMurray J.J.V., Solomon S.D., Inzucchi S.E., Køber L., Kosiborod M.N., Martinez F.A., Ponikowski P., Sabatine M.S., Anand I.S., Bělohlávek J. (2019). Dapagliflozin in Patients with Heart Failure and Reduced Ejection Fraction. N. Engl. J. Med..

[166] Herrington W.G., Staplin N., Wanner C., Green J.B., Hauske S.J., Emberson J.R., Preiss D., Judge P., Mayne K.J., The EMPA-KIDNEY Collaborative Group (2023). Empagliflozin in Patients with Chronic Kidney Disease. N. Engl. J. Med..

[167] Sattar N., Lee M.M.Y., Kristensen S.L., Branch K.R.H., Del Prato S., Khurmi N.S., Lam C.S.P., Lopes R.D., McMurray J.J.V., Pratley R.E. (2021). Cardiovascular, mortality, and kidney outcomes with GLP-1 receptor agonists in patients with type 2 diabetes: A systematic review and meta-analysis of randomised trials. Lancet Diabetes Endocrinol..

[168] Ryan D.H., Lingvay I., Colhoun H.M., Deanfield J., Emerson S.S., Kahn S.E., Kushner R.F., Marso S., Plutzky J., Brown-Frandsen K. (2020). Semaglutide Effects on Cardiovascular Outcomes in People with Overweight or Obesity (SELECT) rationale and design. Am. Heart J..

[169] Packer M., Zile M.R., Kramer C.M., Murakami M., Ou Y., Borlaug B.A., SUMMIT Trial Study Group (2025). Interplay of Chronic Kidney Disease and the Effects of Tirzepatide in Patients with Heart Failure, Preserved Ejection Fraction, and Obesity: The SUMMIT Trial. J. Am. Coll. Cardiol..

[170] González-Juanatey J.R., Górriz J.L., Ortiz A., Valle A., Soler M.J., Facila L. (2023). Cardiorenal benefits of finerenone: Protecting kidney and heart. Ann. Med..

[171] Bakris G.L., Agarwal R., Anker S.D., Pitt B., Ruilope L.M., Rossing P., Kolkhof P., Nowack C., Schloemer P., Joseph A. (2020). Effect of Finerenone on Chronic Kidney Disease Outcomes in Type 2 Diabetes. N. Engl. J. Med..

[172] Agarwal R., Filippatos G., Pitt B., Anker S.D., Rossing P., Joseph A., Kolkhof P., Nowack C., Gebel M., Ruilope L.M. (2022). Cardiovascular and kidney outcomes with finerenone in patients with type 2 diabetes and chronic kidney disease: The FIDELITY pooled analysis. Eur. Heart J..

[173] Guerrero-Mauvecin J., Villar-Gómez N., Miño-Izquierdo L., Povo-Retana A., Ramos A.M., Ruiz-Hurtado G., Sanchez-Niño M.D., Ortiz A., Sanz A.B. (2025). Antioxidant Effects of SGLT2 Inhibitors on Cardiovascular-Kidney-Metabolic (CKM) Syndrome. Antioxidants.

[174] Myasoedova V.A., Bozzi M., Valerio V., Moschetta D., Massaiu I., Rusconi V., Di Napoli D., Ciccarelli M., Parisi V., Agostoni P. (2023). Anti-Inflammation and Anti-Oxidation: The Key to Unlocking the Cardiovascular Potential of SGLT2 Inhibitors and GLP1 Receptor Agonists. Antioxidants.

[175] Luna-Marco C., Iannantuoni F., Hermo-Argibay A., Devos D., Salazar J.D., Víctor V.M., Rovira-Llopis S. (2024). Cardiovascular benefits of SGLT2 inhibitors and GLP-1 receptor agonists through effects on mitochondrial function and oxidative stress. Free. Radic. Biol. Med..

[176] Cowie M.R., Fisher M. (2020). SGLT2 inhibitors: Mechanisms of cardiovascular benefit beyond glycaemic control. Nat. Rev. Cardiol..

[177] Ravid J.D., Laffin L.J. (2022). Effects of Finerenone, a Novel Nonsteroidal Mineralocorticoid Receptor Antagonist, on Cardiovascular Disease, Chronic Kidney Disease, and Blood Pressure. Curr. Cardiol. Rep..

[178] Walton E.L. (2016). The dual role of ROS, antioxidants and autophagy in cancer. Biomed. J..

[179] Harris I.S., DeNicola G.M. (2020). The Complex Interplay between Antioxidants and ROS in Cancer. Trends Cell Biol..

[180] Gluckman P.D., Hanson M.A., Bateson P., Beedle A.S., Law C.M., Bhutta Z.A., Anokhin K.V., Bougnères P., Chandak G.R., Dasgupta P. (2009). Towards a new developmental synthesis: Adaptive developmental plasticity and human disease. Lancet.

